# Walking Speed Explains Apparent Ground Reaction Force Differences in Parkinson’s Disease: A Waveform‐Level SPM Analysis During Normal and Virtual Reality Walking

**DOI:** 10.1155/padi/5474367

**Published:** 2026-07-16

**Authors:** Elaheh Azadian, Mahdi Majlesi, Alireza Ardalany Moghadam, Makwan Jabar Ali, Rezvan Bakhtiarian

**Affiliations:** ^1^ Department of Motor Behavior, Ha.C., Islamic Azad University, Hamedan, Iran, azad.ac.ir; ^2^ Department of Sport Biomechanics, Ha.C., Islamic Azad University, Hamedan, Iran, azad.ac.ir; ^3^ Department of Corrective Exercise & Sport Injury, Faculty of Physical Education and Sport Sciences, Allameh Tabataba’i University, Tehran, Iran, atu.ac.ir; ^4^ Physical Education and Sport Sciences Department, University of Halabja, Halabja, Kurdistan Region, Iraq

**Keywords:** ground reaction force, Parkinson’s disease, statistical parametric mapping, virtual reality, walking speed

## Abstract

**Background:**

Ground reaction force (GRF) alterations are reported in Parkinson’s disease (PD), but most kinetic measures depend on walking speed. Since individuals with PD walk more slowly, apparent differences may reflect speed rather than disease‐specific biomechanics. Virtual reality (VR) may further modify gait kinetics beyond speed effects.

**Objective:**

To determine whether between‐group differences in GRF waveforms during overground walking persist after accounting for walking speed and whether VR induces speed‐independent kinetic modulation in individuals with PD.

**Methods:**

14 adults with mild‐to‐moderate PD (Hoehn and Yahr stage II–III) and seventeen age‐matched controls performed overground walking under normal and immersive VR conditions. Three‐dimensional kinematics (100 Hz) and GRFs (1000 Hz) were recorded. Discrete spatiotemporal and kinetic variables were analyzed using mixed‐design repeated‐measures ANOVA. Time‐continuous GRF waveforms were examined using mixed‐model statistical parametric mapping (SPM) ANCOVA with walking speed entered as a covariate.

**Results:**

Individuals with PD walked significantly slower than controls in both conditions. VR increased stride length and modulated several discrete GRF variables, including anterior–posterior and mediolateral components. However, waveform‐level SPM analyses revealed no persistent group or group × task effects once walking speed was included as a covariate. In contrast, walking speed demonstrated widespread and robust associations with vertical and mediolateral GRF trajectories across multiple stance‐phase intervals.

**Conclusion:**

During comfortable overground walking in mild‐to‐moderate PD, many apparent kinetic differences are largely speed‐coupled rather than clearly speed‐independent. These findings underscore the necessity of speed‐aware modeling when interpreting gait kinetics and evaluating VR‐based paradigms in Parkinson’s disease.

## 1. Introduction

Gait impairment in Parkinson’s disease (PD) is multifactorial and clinically significant, contributing to reduced mobility, loss of independence, and an increased risk of falls [1, 2]. A characteristic gait phenotype is marked by reduced walking speed and shortened step length, frequently accompanied by impaired dynamic balance control and diminished adaptability to environmental demands [3, 4]. From a biomechanical standpoint, kinetic variables—particularly ground reaction forces (GRFs) and the resulting joint moments and powers—are of particular interest because they quantify how neuromuscular control strategies are translated into whole‐body acceleration and weight support during stance. An important methodological challenge in gait biomechanics is that kinetic measures are intrinsically coupled to walking speed [5, 6]. This issue is particularly relevant in PD, where reduced gait speed is a characteristic feature and may substantially influence kinetic outcomes [7, 8]. Consequently, when individuals with PD walk more slowly than controls, observed between‐group differences in GRF peaks or waveforms may represent speed‐related artefacts rather than disease‐specific biomechanical signatures.

The problem of speed‐related confounding is not merely statistical but mechanistic in nature. Walking speed partly reflects the output of basal ganglia–cortical circuitry that governs movement vigor and amplitude scaling. In PD, bradykinesia and impaired amplitude regulation reduce step length and overall gait speed, while rigidity and disrupted intersegmental coordination may further constrain propulsion and compromise dynamic stability [9, 10]. Critically, if reduced walking speed lies on the causal pathway linking PD pathophysiology to altered kinetic patterns, statistical adjustment for speed may remove variance that is mechanistically attributable to the disease process itself. Conversely, failure to account for speed may exaggerate apparent disease effects. A scientifically defensible approach therefore requires explicit testing of whether kinetic differences persist after controlling for speed, with speed‐adjusted findings interpreted as conditional effects rather than unconditional disease‐specific markers [11, 12].

Beyond walking speed, sensory context can acutely modulate gait control in PD. Individuals with PD often demonstrate increased reliance on external sensory cues and altered multisensory reweighting, accompanied by greater attentional demands to maintain stable locomotion [13]. Virtual reality (VR) walking enables controlled manipulation of visual flow, environmental complexity, and perceived threat and has been employed both as an experimental paradigm and as a rehabilitation modality targeting gait and balance in PD [14, 15]. VR may modify step length and variability through mechanisms analogous to external cueing (e.g., augmented optic flow), yet it can also increase cognitive load and destabilize posture depending on task design and individual susceptibility [16]. It remains unclear whether VR‐induced alterations in kinetic profiles occur independently of concomitant changes in walking speed. Furthermore, because walking speed may influence gait kinetics under both conventional and virtual walking conditions, examining only VR‐related changes without reference to normal walking may obscure whether observed kinetic differences reflect sensory‐context effects, speed‐related adaptations, or a combination of both. Simultaneous evaluation of normal and VR walking is therefore necessary to determine whether the influence of walking speed on gait kinetics differs across sensory environments.

A further limitation of the PD gait kinetics literature is its predominant reliance on discrete scalar outcomes (e.g., peak vertical GRF), which may fail to detect temporally localized effects and are often sensitive to feature definition and measurement noise. Waveform‐based inferential approaches, such as statistical parametric mapping (SPM), enable hypothesis testing across the entire time‐normalized trajectory while controlling family‐wise error rates under the assumptions of random field theory [17, 18]. In gait biomechanics, SPM has been increasingly applied to detect phase‐specific differences in kinematics, kinetics, and electromyography that are not captured by peak‐based metrics [19, 20]. Importantly, SPM frameworks permit the inclusion of covariates (e.g., walking speed) within the general linear model at each time point, allowing direct evaluation of whether group‐related waveform differences persist after accounting for speed.

The present study was designed to determine whether kinetic differences observed between individuals with PD and healthy age‐matched controls persist after accounting for walking speed and whether immersive VR induces gait kinetic adaptations beyond those attributable to speed alone. The inclusion of a healthy control group enabled differentiation between disease‐related effects and general responses to VR exposure. To address these questions, we analyzed both discrete GRF features and time‐continuous GRF waveforms using mixed‐model repeated‐measures ANOVA and SPM‐based ANCOVA, with walking speed entered as a covariate within the general linear model during normal overground and VR walking. Based on previous evidence demonstrating slower gait and altered force‐generation patterns in PD, we hypothesized that (i) individuals with PD would walk more slowly than healthy controls in both walking conditions; (ii) VR would modify spatiotemporal gait characteristics and GRF profiles in both groups; and (iii) apparent between‐group differences in GRF waveforms would be reduced after adjustment for walking speed, indicating that a substantial proportion of the observed kinetic variance is associated with gait speed rather than disease status alone.

## 2. Methods

### 2.1. Participants

An a priori sample size calculation was performed using G × Power software (Version 3.1) for a repeated‐measures ANOVA design. A large effect size (*f* = 0.40) was assumed according to Cohen’s conventional criteria for behavioral and clinical research, with the significance level set at *α* = 0.05 and the desired statistical power (1−β) set at 0.80. The analysis indicated a minimum required sample size of 12 participants per group. Thirty‐one individuals were ultimately enrolled, including 14 adults with idiopathic PD and 17 neurologically healthy age‐matched controls. PD diagnosis was confirmed by an experienced neurologist [21]. Participants were recruited from Hamedan Province through community outreach, outpatient clinics, and Besat Hospital. Eligibility criteria for the PD group included Hoehn and Yahr stage II–III (mean stage 2.5 ± 0.5) [22], assessment during the ON‐medication state, independent ambulation without assistive devices, and adequate vision and hearing for safe VR exposure. Exclusion criteria comprised atypical or secondary parkinsonism, lower‐limb surgery within the previous year, implanted medical devices incompatible with motion capture, severe musculoskeletal disorders affecting gait, use of psychoactive medications other than standard PD treatment, and clinically significant cognitive impairment. All participants were aged between 50 and 70 years. The study protocol was approved by the Ethics Committee of Islamic Azad University, Hamedan Branch (IR.IAU.H.REC.1401.001), and was conducted in accordance with the Declaration of Helsinki. Written informed consent was obtained from all participants prior to testing.

### 2.2. Instruments and Examination

Lower‐extremity three‐dimensional kinematics were recorded using a six‐camera optoelectronic motion capture system operating at 100 Hz (Vicon, Oxford Metrics, Oxford, UK). Participants walked barefoot, and 16 retroreflective markers were affixed in accordance with the Plug‐in Gait lower‐limb biomechanical model [21, 23]. GRFs were obtained at 1000 Hz from two floor‐mounted force platforms (Type 9281, Kistler Instrument AG, Winterthur, Switzerland). Force signals were acquired along the vertical (Fz), anterior–posterior (Fy), and mediolateral (Fx) directions. Kinematic and kinetic data streams were synchronized through the laboratory acquisition interface to ensure temporal alignment.

Data were collected during overground walking along a 12‐m walkway under two experimental conditions: (i) normal gait (NG) and (ii) VR gait (VRG). Where possible, task order was counterbalanced to minimize systematic learning or fatigue effects. Before formal recording, participants completed familiarization trials to accustom themselves to the walkway, foot placement relative to the force plates, and—during the VR condition—the immersive virtual environment. To preserve ecological validity within the VR task, a digital replica of the laboratory layout, including force‐plate locations, was developed in Unity (Unity Technologies, USA) and presented via an Oculus Quest head‐mounted display (Facebook Technologies Ltd., USA). The virtual environment was presented using an Oculus Quest 2 head‐mounted display (Meta Platforms Inc., USA), providing a refresh rate of 90 Hz and an approximate field of view of 110°. Head movements were tracked using the headset’s built‐in six‐degrees‐of‐freedom (6DoF) inside‐out tracking system [24]. To ensure spatial correspondence between the virtual and physical environments, the laboratory layout, including the walkway dimensions and force‐plate locations, was replicated in the Unity‐based virtual environment [25]. To ensure consistency between the real and virtual environments, the dimensions of the laboratory walkway were reproduced in Unity based on direct measurements of the experimental space. The location of the force plates and the start and end points of the walking path were represented at corresponding positions within the virtual environment. Participants performed the walking task along the same physical walkway in both conditions, while the virtual scene provided a visually matched representation of the laboratory layout. In each condition, six walking trials were performed at a self‐selected comfortable speed. Trials affected by technical artefacts (e.g., brief marker occlusion or atypical force‐plate contact) were discarded. From the remaining valid recordings, three trials demonstrating the highest signal quality were retained for subsequent analysis for each participant and condition.

GRF data were processed using a fourth‐order zero‐lag low‐pass Butterworth filter with a cut‐off frequency of 20 Hz [26]. To facilitate interindividual comparison, all force signals were normalized to body weight. Initial contact and toe‐off were identified using a vertical GRF threshold of 20 N, consistent with commonly used force‐based gait event detection procedures [27] and previous biomechanical studies employing GRF thresholds for gait event identification. The stance phase for each limb was defined as the interval between these two gait events, and GRF waveforms were subsequently resampled to a standardized 0%–100% stance interval to enable time‐continuous analysis. Spatiotemporal parameters comprised walking speed and stride length, calculated from the kinematic trajectories and averaged across the retained trials for each task and limb. Discrete kinetic variables were defined a priori according to established biomechanical conventions and prior laboratory protocols. The vertical GRF, characterized by its typical bimodal stance pattern, was quantified using the first loading peak (Zp1), the mid‐stance trough (Zp2), and the terminal push‐off peak (Zp3), when present. For the anterior–posterior component, peak braking (Yp1) and peak propulsive force (Yp2) were extracted. The mediolateral force profile was represented by the principal early‐stance peak (Xp1). Vertical loading rate was computed as the slope of the vertical GRF from initial contact to the first loading peak [28]. For each participant and experimental condition, discrete outcomes were averaged across the three highest‐quality trials. Data from the right and left limbs were retained separately to allow explicit modeling of within‐subject repeated measures across limbs.

### 2.3. Statistical Analysis

All statistical analyses were performed using SPSS (Version 26, IBM Corp., Armonk, NY, USA), with the significance threshold set at *α* = 0.05. Assumptions of parametric testing were evaluated at the model level. Normality of residuals and homogeneity of variance were assessed using visual inspection of diagnostic plots and standard statistical tests. When the assumption of sphericity was violated in repeated‐measures designs, Greenhouse–Geisser corrections were applied. Discrete variables—including walking speed, stride length, GRF peak magnitudes, and vertical loading rate—were analyzed using mixed‐design repeated‐measures ANOVA. Group (PD vs. control) was specified as a between‐subject factor, while task (normal overground vs. VR) and foot (right vs. left) were treated as within‐subject factors. Inclusion of foot as a repeated‐measures factor allowed potential gait asymmetries and laterality‐related effects, which are common in PD, to be evaluated within the statistical model. Significant interactions were followed by planned simple‐effects analyses, comprising within‐group task comparisons and between‐group contrasts within each task condition. Time‐continuous waveform analyses were conducted using SPM within a general linear model framework implemented in the Python‐based spm1d package (Version 0.4.53). Mixed‐model SPM ANCOVA was performed using the spm1d.stats.glm function, with walking speed entered as a continuous covariate. Separate models were estimated for each GRF component (Fx, Fy, Fz) and for each limb. Statistical inference was performed across the full time‐normalized stance waveform, with family‐wise error rate controlled according to random field theory at *α* = 0.05. Significant supra‐threshold clusters were identified and interpreted as temporally localized effects. This modeling strategy enabled evaluation of the main effects of group and task, their interaction, and the contribution of walking speed to GRF trajectory variance. ANOVA results are reported as F(df1, df2) = value, *p* = value, and partial eta squared (ηp^2^), where df1 and df2 represent the numerator and denominator degrees of freedom, respectively. Effect sizes for all ANOVA effects were quantified using partial eta squared (ηp^2^).

## 3. Results

Table 1 displays the demographic characteristics of the participants, as well as a comparison of these characteristics between the two groups. Table 2 presents the omnibus mixed‐design ANOVA results for all spatiotemporal and GRF variables. When significant main effects or interactions were identified, planned simple‐effects analyses were performed to examine between‐group differences within each walking condition and within‐group differences between walking conditions. The corresponding simple‐effects statistics are reported in the text.

**TABLE 1:** Demographic and clinical characteristics of participants in the Parkinson’s disease (PD) and control groups.

	Groups		*p* value
	PD	Control	
N (female, male)	14 (7,7)	17 (8,9)	
Age (year)	61.60 ± 6.23	60.52 ± 6.17	0.622
Mass (Kg)	67.60 ± 10.56	68.88 ± 11.60	0.751
Height (cm)	1.64 ± 0.10	1.64 ± 0.09	0.864
BMI (kg/m^2^)	25.13 ± 3.39	25.71 ± 3.45	0.641
Disease duration (year)	6.50 ± 0.90	—	—
Hoehn and Yahr stage	2.50 ± 0.50	—	—

*Note:* Values are presented as mean ± standard deviation unless otherwise stated. *p* values reflect between‐group comparisons (independent samples *t*‐test). Clinical variables are reported for the PD group only.

Abbreviations: BMI, body mass index; PD, Parkinson’s disease.

**TABLE 2:** Descriptive statistics and mixed‐design ANOVA results for spatiotemporal and ground reaction force (GRF) variables.

Variables	Foot	PD		Control		Group	Task	Foot	Group × task	Group × foot
		Normal gait	VR gait	Normal gait	VR gait					
Speed (m/s)	Right	0.79 ± 0.26	0.82 ± 0.31	1.02 ± 0.16	1.04 ± 0.18	*F* = 7.951	*F* = 1.170	*F* = 0.642	*F* = 0.001	*F* = 0.095
	Left	0.79 ± 0.25	0.81 ± 0.30	1.01 ± 0.16	1.03 ± 0.20	*p* = **0.008** ηp^2^ = 0.210	*p* = 0.288 ηp^2^ = 0.037	*p* = 0.429 ηp^2^ = 0.021	*p* = 0.970 ηp^2^ = 0.000	*p* = 0.760 ηp^2^ = 0.003

Stride length (m)	Right	1.03 ± 0.23	1.10 ± 0.32	1.14 ± 0.11	1.24 ± 0.13	*F* = 3.474	*F* = 11.780	*F* = 0.207	*F* = 0.545	*F* = 0.026
	Left	1.03 ± 0.22	1.09 ± 0.29	1.13 ± 0.11	1.24 ± 0.14	*p* = 0.072 ηp^2^ = 0.104	*p* = **0.002** ηp^2^ = 0.282	*p* = 0.653 ηp^2^ = 0.007	*p* = 0.466 ηp^2^ = 0.018	*p* = 0.873 ηp^2^ = 0.001

Zp_1_ (BW)	Right	1.05 ± 0.07	1.14 ± 0.16	1.07 ± 0.12	1.09 ± 0.15	*F* = 0.118	*F* = 6.030	*F* = 0.001	*F* = 0.577	*F* = 0.072
	Left	1.07 ± 0.08	1.12 ± 0.13	1.06 ± 0.12	1.11 ± 0.12	*p* = 0.733 ηp^2^ = 0.004	*p* = **0.020** ηp^2^ = 0.172	*p* = 0.972 ηp^2^ = 0.000	*p* = 0.454 ηp^2^ = 0.020	*p* = 0.791 ηp^2^ = 0.002

Zp_2_ (BW)	Right	0.91 ± 0.07	0.94 ± 0.14	0.86 ± 0.05	0.84 ± 0.08	*F* = 7.472	*F* = 0.105	*F* = 3.630	*F* = 1.459	*F* = 0.576
	Left	0.91 ± 0.07	0.91 ± 0.13	0.85 ± 0.06	0.83 ± 0.07	*p* = **0.011** ηp^2^ = 0.205	*p* = 0.748 ηp^2^ = 0.004	*p* = 0.067 ηp^2^ = 0.111	*p* = 0.210 ηp^2^ = 0.054	*p* = 0.454 ηp^2^ = 0.019

Zp_3_ (BW)	Right	1.07 ± 0.07	1.15 ± 0.17	1.07 ± 0.06	1.09 ± 0.07	*F* = 0.935	*F* = 1.870	*F* = 1.980	*F* = 1.887	*F* = 0.214
	Left	1.07 ± 0.05	1.12 ± 0.15	1.08 ± 0.08	1.07 ± 0.08	*p* = 0.342 ηp^2^ = 0.031	*p* = 0.182 ηp^2^ = 0.061	*p* = 0.170 ηp^2^ = 0.064	*p* = 0.179 ηp^2^ = 0.061	*p* = 0.647 ηp^2^ = 0.007

Yp_1_ (BW)	Right	0.24 ± 0.04	0.37 ± 0.10	0.28 ± 0.05	0.36 ± 0.09	*F* = 0.075	*F* = 51.530	*F* = 1.986	*F* = 3.800	*F* = 3.587
	Left	0.27 ± 0.07	0.40 ± 0.11	0.28 ± 0.06	0.35 ± 0.08	*p* = 0.787 ηp^2^ = 0.003	***p* < 0.001** ηp^2^ = 0.640	*p* = 0.169 ηp^2^ = 0.064	*p* = 0.061 ηp^2^ = 0.116	*p* = 0.068 ηp^2^ = 0.110

Yp_2_ (BW)	Right	0.31 ± 0.06	0.40 ± 0.09	0.34 ± 0.05	0.36 ± 0.08	*F* = 0.465	*F* = 18.570	*F* = 2.670	*F* = 5.575	*F* = 3.379
	Left	0.29 ± 0.06	0.36 ± 0.07	0.34 ± 0.06	0.37 ± 0.07	*p* = 0.501 ηp^2^ = 0.016	***p* < 0.001** ηp^2^ = 0.390	*p* = 0.113 ηp^2^ = 0.084	*p* = **0.025** ηp^2^ = 0.161	*p* = 0.076 ηp^2^ = 0.104

Xp_1_ (BW)	Right	0.03 ± 0.01	0.06 ± 0.03	0.05 ± 0.02	0.09 ± 0.05	*F* = 9.390	*F* = 29.070	*F* = 0.974	*F* = 1.049	*F* = 2.986
	Left	0.03 ± 0.01	0.06 ± 0.01	0.06 ± 0.01	0.10 ± 0.05	*p* = **0.005** ηp^2^ = 0.245	***p* < 0.001** ηp^2^ = 0.501	*p* = 0.332 ηp^2^ = 0.032	*p* = 0.315 ηp^2^ = 0.035	*p* = 0.094 ηp^2^ = 0.094

Loading rate (BW/s)	Right	30.59 ± 13.39	37.73 ± 8.92	37.55 ± 9.45	42.28 ± 13.08	*F* = 2.085	*F* = 12.250	*F* = 3.885	*F* = 0.276	*F* = 0.120
	Left	32.63 ± 13.30	39.91 ± 10.23	38.43 ± 10.83	44.36 ± 15.31	*p* = 0.159 ηp^2^ = 0.067	*p* = **0.002** ηp^2^ = 0.297	*p* = 0.058 ηp^2^ = 0.118	*p* = 0.603 ηp^2^ = 0.009	*p* = 0.732 ηp^2^ = 0.004

*Note:* Values are presented as mean ± standard deviation. ANOVA results represent mixed‐design repeated‐measures analyses with Group (PD vs. Control) as a between‐subject factor and Task (Normal gait vs. VR) and Foot (Right vs. Left) as within‐subject factors. Bold values indicate statistically significant effects (*p* < 0.05). Walking speed is expressed in m/s, stride length in meters (m), GRF peak variables (Zp1, Zp2, Zp3, Yp1, Yp2, and Xp1) are normalized to body weight (BW), and loading rate is expressed in BW/s. All F statistics are reported with numerator and denominator degrees of freedom of (1, 29).

Abbreviations: BW, body weight; GRF, ground reaction force; PD, Parkinson’s disease; VR, virtual reality.

A significant main effect of group was observed for walking speed, with individuals with PD walking slower than controls in both NG (*F*(1,29) = 8.93, *p* = 0.006, ηp^2^ = 0.23) and VR (*F*(1,29) = 6.23, *p* = 0.018, ηp^2^ = 0.17) conditions. VR did not significantly alter walking speed in either group (*p* > 0.05). Stride length showed a significant main effect of task, increasing during VR, with significance reached in controls (*F*(1,29) = 9.28, *p* = 0.005, ηp^2^ = 0.24) and approaching significance in PD (*F*(1,29) = 3.41, *p* = 0.074, ηp^2^ = 0.10). For the vertical component, the first peak (Zp1) demonstrated a significant task effect, with an approximately 5% reduction during VR; this decrease was significant in PD (*F*(1,29) = 4.71, *p* = 0.038, ηp^2^ = 0.14) but not in controls. The second vertical peak (Zp2) showed a significant main effect of group (*F*(1,29) = 7.47, *p* = 0.011, ηp^2^ = 0.21), with PD exhibiting ∼9% higher values than controls across conditions. Between‐group differences were significant in both NG (*F*(1,29) = 6.74, *p* = 0.015, ηp^2^ = 0.19) and VR (*F*(1,29) = 6.16, *p* = 0.016, ηp^2^ = 0.18), while within‐group condition effects were not significant. No significant effects were detected for Zp3. In the anterior–posterior component, Yp1 increased significantly during VR in both PD (*F*(1,29) = 37.99, *p* < 0.001, ηp^2^ = 0.57) and controls (*F*(1,29) = 15.13, *p* = 0.001, ηp^2^ = 0.34), without between‐group differences. For Yp2, both a significant task effect and a group × task interaction were found. VR selectively increased Yp2 in PD (*F*(1,29) = 20.30, *p* < 0.001, ηp^2^ = 0.41), whereas no significant change occurred in controls. A between‐group difference was present during NG (*F*(1,29) = 5.03, *p* = 0.033, ηp^2^ = 0.15) but disappeared under VR. In the mediolateral component (Fx), significant main effects of group and task were observed. Controls exhibited greater mediolateral forces than PD in NG (*F*(1,29) = 15.45, *p* < 0.001, ηp^2^ = 0.35) and VR (*F*(1,29) = 5.16, *p* = 0.031, ηp^2^ = 0.15), and VR increased Fx in both groups. Loading rate also showed a significant task effect, increasing during VR in both PD (*F*(1,29) = 7.39, *p* = 0.011, ηp^2^ = 0.20) and controls (*F*(1,29) = 4.90, *p* = 0.035, ηp^2^ = 0.15), without a group effect (Table 2). No significant main effects of foot or group × foot interactions were observed for any of the discrete GRF variables (all *p* > 0.05), indicating comparable kinetic patterns between the right and left limbs in both groups.

**FIGURE 1:**
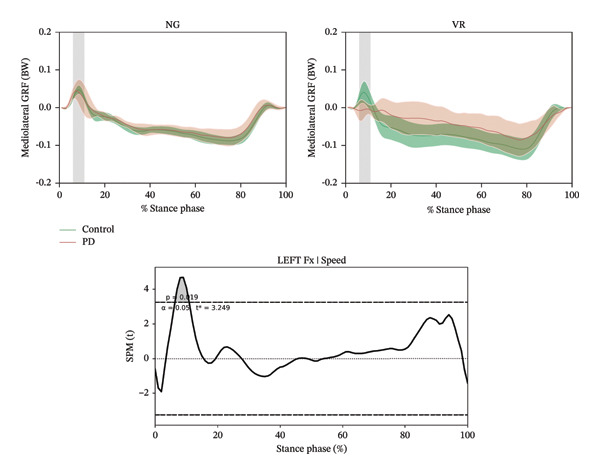
Left‐limb mediolateral ground reaction force waveforms during normal and virtual‐reality walking and waveform‐level SPM ANCOVA results. Mean (±SD) mediolateral ground reaction force (Fx), normalized to body weight (BW), across the stance phase during normal gait (NG) and virtual‐reality gait (VR) in individuals with Parkinson’s disease (PD) and healthy controls (top panels). The lower panel presents the statistical parametric mapping (SPM{t}) trajectory derived from the mixed‐model SPM ANCOVA examining the effect of walking speed as a covariate on the Fx waveform. Dashed horizontal lines indicate the critical random‐field‐theory threshold for statistical significance (*α* = 0.05), and supra‐threshold clusters identify stance‐phase intervals in which walking speed significantly influenced the mediolateral GRF trajectory. Shaded regions in the waveform panels correspond to stance‐phase intervals identified as significant in the corresponding waveform‐level SPM analyses.

**FIGURE 2:**
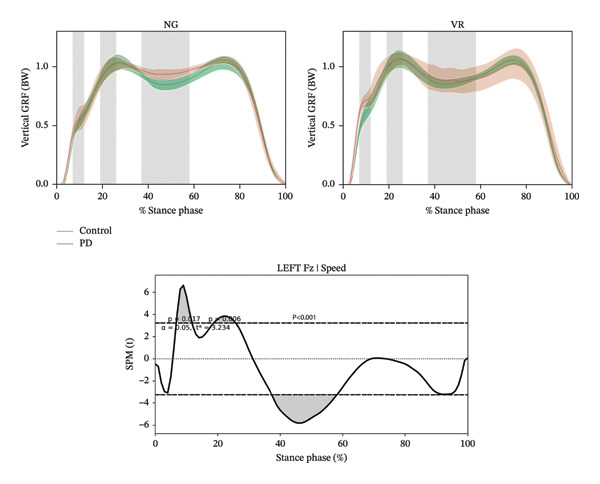
Left‐limb vertical ground reaction force waveforms during normal and virtual‐reality walking and waveform‐level SPM ANCOVA results. Mean (±SD) vertical ground reaction force (Fz), normalized to body weight (BW), across the stance phase during normal gait (NG) and virtual‐reality gait (VR) in individuals with Parkinson’s disease (PD) and healthy controls (top panels). The lower panel presents the statistical parametric mapping (SPM{t}) trajectory derived from the mixed‐model SPM ANCOVA examining the effect of walking speed as a covariate on the Fz waveform. Dashed horizontal lines indicate the critical random‐field‐theory threshold for statistical significance (*α* = 0.05), and supra‐threshold clusters (shaded regions) identify stance‐phase intervals in which walking speed significantly influenced the vertical GRF trajectory. Shaded regions in the waveform panels correspond to stance‐phase intervals identified as significant in the corresponding waveform‐level SPM analyses.

**FIGURE 3:**
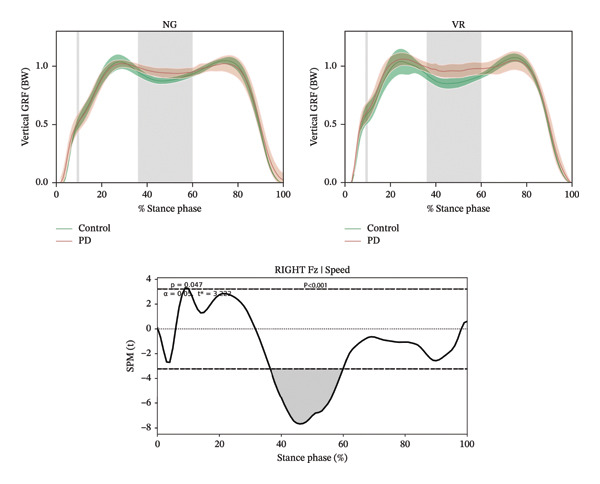
Right‐limb vertical ground reaction force waveforms during normal and virtual‐reality walking and waveform‐level SPM ANCOVA results. Mean (±SD) vertical ground reaction force (Fz), normalized to body weight (BW), across the stance phase during normal gait (NG) and virtual‐reality gait (VR) in individuals with Parkinson’s disease (PD) and healthy controls (top panels). The lower panel presents the statistical parametric mapping (SPM{t}) trajectory derived from the mixed‐model SPM ANCOVA examining the effect of walking speed as a covariate on the Fz waveform. Dashed horizontal lines indicate the critical random‐field‐theory threshold for statistical significance (*α* = 0.05), and supra‐threshold clusters identify stance‐phase intervals in which walking speed significantly influenced the vertical GRF trajectory. Shaded regions in the waveform panels correspond to stance‐phase intervals identified as significant in the corresponding waveform‐level SPM analyses.

Waveform‐level SPM ANCOVA results are illustrated in Figures 1–3. Mixed‐model SPM ANCOVA revealed no significant main effects of group or group × task interactions in any GRF component at the waveform level. In all nonsignificant comparisons, the SPM test statistic trajectories remained below the critical random‐field‐theory threshold throughout the stance phase, and no supra‐threshold clusters were identified. A localized main effect of task was detected in the right mediolateral component (Fx) during early stance (6.7%–11.0% gait cycle, *p* = 0.021), indicating temporally restricted VR‐related modulation. In contrast, walking speed demonstrated significant effects across multiple stance‐phase regions. In the left mediolateral component (Fx), walking speed significantly influenced the waveform during early stance (6.3%–10.9%, *p* = 0.019) (Figure 1). Similarly, in the left vertical component (Fz), speed significantly affected the waveform across early (6.7%–11.8%, *p* = 0.017), mid (18.7%–25.7%, *p* = 0.006), and late stance (37.2%–58.2%, *p* < 0.001) phases of stance (Figure 2). In the right vertical component (Fz), speed influenced both early stance (8.8%–10.1%, *p* = 0.047) and a substantial mid‐to late‐stance interval (36.4%–59.9%, *p* < 0.001) (Figure 3). Importantly, once walking speed was included as a covariate, no persistent between‐group waveform differences were observed.

## 4. Discussion

This study investigated whether kinetic differences in PD persist after accounting for walking speed and whether VR walking produces additional kinetic modulation beyond speed‐related effects. Waveform‐level analyses showed that, after adjusting for walking speed, no sustained group or group × task effects remained across the GRF waveforms. In contrast, walking speed demonstrated widespread and statistically robust associations with vertical and mediolateral GRF trajectories across multiple phases of stance. These findings suggest that many between‐group kinetic differences are substantially attenuated once speed is modeled, underscoring the importance of speed‐aware analysis when interpreting GRF alterations in PD [29].

Individuals with PD walked more slowly than controls in both NG and VR, and VR did not significantly change walking speed in either group. Stride length increased during VR—clearly in controls and marginally in PD. Reduced preferred speed is a well‐established feature of PD, commonly attributed to impaired movement scaling and diminished gait automaticity [30]. Visually enriched environments can increase step length through external cueing mechanisms, although effects depend on scene characteristics and cognitive load [31, 32]. The increase in stride length without a corresponding rise in speed suggests compensatory adjustments in cadence or stance duration, consistent with a cautious stability strategy under altered sensory conditions. In PD, impaired step‐length regulation reflects basal ganglia dysfunction affecting amplitude control and internal timing [33]. Visual input may provide an external spatial reference that facilitates larger steps; however, preserved speed despite longer strides implies concurrent regulatory adjustments, potentially involving cadence modulation or increased attentional demand [13, 30, 34]. Clinically, this dissociation indicates that VR can modify spatiotemporal gait patterns without altering overall walking speed.

For the discrete outcomes, Zp1 was reduced by approximately 5% during VR, reaching statistical significance in the PD group. Zp2 showed a clear group effect, with PD demonstrating roughly 9% higher values than controls across both tasks, whereas Zp3 showed no significant effects. Vertical GRF characteristics are known to be influenced by walking speed, with slower walking generally associated with lower peak forces and altered loading patterns [27]. In the present study, the higher Zp2 values observed in PD indicate a less pronounced mid‐stance trough despite slower walking. Because Zp2 represents the mid‐stance minimum of the vertical GRF curve rather than the terminal push‐off peak, this finding may reflect altered body‐weight transfer, reduced vertical center‐of‐mass excursion, or modified support‐phase mechanics during single‐limb stance. However, because the present study did not include inverse dynamics analyses, the specific joint‐level contributions underlying these GRF alterations cannot be determined directly. Furthermore, waveform‐level analyses demonstrated that the observed group differences did not persist after adjustment for walking speed, suggesting that these discrete differences should be interpreted cautiously and may be partly attributable to speed‐related influences rather than robust disease‐specific kinetic alterations.

The reduction in Zp1 during VR—significant in the PD group—may reflect a more cautious loading strategy under altered sensory conditions, potentially related to increased attentional demands or perceived postural threat [13, 35]. However, waveform‐level analyses demonstrated that these group‐related differences did not persist after adjustment for walking speed, indicating that the observed discrete effects should be interpreted cautiously. This finding highlights the substantial influence of walking speed on vertical GRF characteristics and suggests that apparent between‐group kinetic differences may be partly attributable to speed‐related adaptations rather than unequivocal disease‐specific biomechanical alterations. Accordingly, within the range of walking speeds observed in the present sample, no robust speed‐independent group differences were identified in the GRF waveforms.

VR increased peak braking force (Yp1) in both groups without between‐group differences. For peak propulsion (Yp2), VR selectively increased values in PD, eliminating the between‐group difference observed during NG. Anterior–posterior GRFs are closely coupled to walking speed and step strategy, with greater braking and propulsion typically supporting faster or longer steps [36]. Reduced propulsive force is commonly reported in PD and has been associated with impaired propulsion and altered interlimb coordination in previous studies [37]. However, because the present study assessed only GRF variables, the specific joint‐level mechanisms underlying these changes cannot be determined directly. The VR‐related increase in Yp2 in PD is therefore consistent with a cueing effect, whereby enhanced visual input may facilitate greater propulsive force production during walking. The absence of a between‐group difference in VR suggests partial normalization of propulsion under enriched sensory conditions. External cues have been shown to improve gait performance in PD by enhancing attentional focus and providing additional sensory information for movement regulation [13, 25, 31]. Biomechanically, longer strides during VR would necessitate greater early braking and late propulsion to redirect the center of mass within each step. The selective increase in Yp2 in PD aligns with evidence that individuals with PD show heightened responsiveness to exteroceptive cueing when internal scaling mechanisms are compromised [34].

Controls generated greater mediolateral GRFs than PD in both tasks, and VR increased mediolateral force in both groups. Loading rate also increased during VR, without a significant group effect. Mediolateral GRFs are associated with frontal‐plane balance control and regulation of the center of mass over the base of support [38]. Increased sensory uncertainty or perceived instability can elevate frontal‐plane control demands, sometimes resulting in greater mediolateral forces or increased cocontraction as stabilization strategies [39]. The consistent VR‐related increases in Fx and loading rate may reflect modifications in gait control during weight acceptance. Although increased loading rates have been associated with stiffer lower‐limb behavior in some gait studies, the present data do not permit direct inference regarding limb stiffness or underlying neuromuscular mechanisms [40, 41]. The lower mediolateral forces observed in PD may reflect reduced dynamic balance responses, diminished lateral weight transfer, or an overall conservative gait strategy. However, given the strong influence of walking speed on GRF magnitude and timing—and the slower speed in PD—part of this difference may be secondary to speed rather than a distinct disease‐specific effect. The elevated loading rate observed during VR reflects more rapid force application during early stance. Increased loading rates have previously been associated with altered lower‐limb stiffness characteristics during locomotion [40, 41]. However, because lower‐limb stiffness and neuromuscular activity were not directly assessed in the present study, the underlying mechanisms cannot be determined from the current data. In PD, altered neuromuscular control and movement regulation may influence how external sensory information affects loading patterns during gait [42]. Clinically, higher loading rates may indicate increased mechanical demands during weight acceptance, although the functional significance of these changes requires further investigation.

It should be noted that discrete and waveform‐level analyses address related but not identical aspects of gait kinetics. Whereas discrete variables quantify predefined biomechanical events, SPM evaluates the entire GRF trajectory and is sensitive to temporally localized waveform differences. Waveform‐level SPM ANCOVA revealed no significant group or group × task effects for any GRF component once walking speed was included as a covariate. A small, localized task effect was observed in right‐limb mediolateral GRF during early stance. In contrast, walking speed emerged as a significant covariate influencing vertical GRF and, to a lesser extent, mediolateral GRF across multiple stance‐phase intervals in the waveform‐level SPM ANCOVA. No sustained between‐group waveform differences persisted after speed adjustment. The predominance of speed effects is biomechanically expected, as walking speed influences center‐of‐mass acceleration, step‐to‐step transitions, stance timing, and vertical loading patterns [43]. The principal inference is that, within this dataset, GRF waveform differences between groups were not robust beyond speed‐related variance. Methodologically, this underscores the importance of speed‐aware modeling in PD gait research; failure to match, stratify, or statistically account for walking speed risks attributing speed‐driven effects to disease status. At the same time, covariate adjustment requires conceptual caution. If reduced walking speed lies on the causal pathway between PD pathology and kinetic alterations, statistical control may attenuate clinically meaningful variance [42, 44]. A balanced interpretation is therefore warranted: GRF differences in PD may be present, but in moderate disease during comfortable walking, they appear largely intertwined with reduced walking speed rather than representing speed‐independent kinetic signatures.

An additional finding was the absence of significant foot effects or group × foot interactions across the analyzed GRF variables. This suggests that, at the level of the measured kinetic outcomes, individuals with PD did not exhibit greater right–left asymmetry than healthy controls. Although motor asymmetry is a well‐recognized clinical feature of PD, the present findings indicate that such asymmetry was not reflected in the analyzed GRF measures under the current experimental conditions. This observation suggests that whole‐body kinetic measures may be less sensitive to subtle laterality‐related gait alterations than clinical motor assessments or joint‐level biomechanical analyses.

This study has several limitations. First, although the sample size was determined a priori using G × Power and was adequate for the planned repeated‐measures analyses, it may have been underpowered to detect subtle effects or interactions at the waveform level using SPM. Future studies employing one‐dimensional biomechanical analyses may benefit from sample size estimation approaches specifically developed for SPM, such as Power1D. Second, the analysis was restricted to GRF variables. Although GRFs provide valuable information regarding overall gait kinetics, they do not allow direct quantification of the contributions of individual joints or muscle groups to braking and propulsion mechanisms. Consequently, interpretations regarding joint‐level biomechanical mechanisms should be considered indirect. Future studies incorporating inverse dynamics analyses, including joint moments and joint power measures, are warranted to provide a more comprehensive understanding of gait kinetics in PD and during VR walking. Third, although walking speed was modeled as a covariate, speed reduction in PD may lie on the causal pathway linking pathology to kinetic alterations; therefore, speed‐adjusted findings should be interpreted as conditional rather than evidence of the complete absence of disease‐related effects. Finally, VR parameters were not systematically manipulated, which may limit generalizability to other immersive environments.

## 5. Conclusion

In summary, individuals with PD walked more slowly than age‐matched controls under both normal and virtual‐reality conditions, and VR modulated several discrete spatiotemporal and kinetic measures. However, waveform‐level mixed‐model SPM analyses showed that, after accounting for walking speed, no sustained between‐group differences persisted across GRF trajectories, whereas walking speed exerted widespread effects on vertical and mediolateral force profiles throughout stance. These results suggest that, during comfortable overground walking in mild‐to‐moderate PD, many apparent kinetic alterations are predominantly speed‐coupled rather than unequivocally speed‐independent biomechanical signatures. Accordingly, rigorous speed‐aware modeling—via matching, stratification, or covariate‐based approaches—is essential for valid interpretation of gait kinetics in PD. Future work should integrate joint‐level kinetics and mediation frameworks to clarify the mechanistic role of walking speed across disease stages and task demands.

## Author Contributions

Elaheh Azadian and Mahdi Majlesi contributed substantially to the study conception and design, data analysis and interpretation, and manuscript drafting. Alireza Ardalany Moghadam was responsible for data extraction and preprocessing. Rezvan Bakhtiarian conducted data collection and experimental testing. Makwan Jabar Ali contributed to critical revision of the manuscript for important intellectual content. All authors reviewed the manuscript and provided feedback.

## Funding

No funding was received for this manuscript.

## Disclosure

All authors approved the final version for publication.

## Conflicts of Interest

The authors declare no conflicts of interest.

## Data Availability

The data that support the findings of this study are available from the corresponding author upon reasonable request.
